# Dynamics of a vibration-driven single disk

**DOI:** 10.1038/s41598-021-95672-6

**Published:** 2021-08-16

**Authors:** Liyang Guan, Li Tian, Meiying Hou, Yilong Han

**Affiliations:** 1 Department of Physics, Hong Kong University of Science and Technology, Clear Water Bay, Hong Kong China; 2 Fachbereich Physik, Universität Konstanz, 78464 Konstanz, Germany; 3 Key Laboratory of Soft Matter Physics, Beijing National Laboratory for Condensed Matter Physics, Institute of Physics, Chinese Academy of Sciences, Beijing, 100190 China; 4 College of Physics, University of Chinese Academy of Sciences, Beijing, 100049 China; 5 The Hong Kong University of Science and Technology Shenzhen Research Institute, Shenzhen, 518057 China

**Keywords:** Soft materials, Statistical physics, thermodynamics and nonlinear dynamics

## Abstract

Granular particles exhibit rich collective behaviors on vibration beds, but the motion of an isolated particle is not well understood even for uniform particles with a simple shape such as disks or spheres. Here we measured the motion of a single disk confined to a quasi-two-dimensional horizontal box on a vertically vibrating stage. The translational displacements obey compressed exponential distributions whose exponent $\beta$ increases with the frequency, while the rotational displacements exhibit unimodal distributions at low frequencies and bimodal distributions at high frequencies. During short time intervals, the translational displacements are subdiffusive and negatively correlated, while the rotational displacements are superdiffusive and positively correlated. After prolonged periods, the rotational displacements become diffusive and their correlations decay to zero. Both the rotational and the translational displacements exhibit white noise at low frequencies, and blue noise for translational motions and Brownian noise for rotational motions at high frequencies. The translational kinetic energy obeys Boltzmann distribution while the rotational kinetic energy deviates from it. Most energy is distributed in translational motions at low frequencies and in rotational motions at high frequencies, which violates the equipartition theorem. Translational and rotational motions are not correlated. These experimental results show that the random diffusion of such driven particles is distinct from thermal motion in both the translational and rotational degrees of freedom, which poses new challenges to theory. The results cast new light on the motion of individual particles and the collective motion of driven granular particles.

## Introduction

Driven granular matter has a broad range of applications. For example, granular particles driven on a vibration or air-blowing bed have been used for ore and powder separation and pattern formation^1^ and non-equilibrium physics studies^2^. Dense granular particles exhibit subdiffusion under shaking^3^ and rotation^4,5^, and levy flights when drained from a silo^6^. Particle motions in dilute granular gases have been studied by experiments in microgravity^7–9^, on a vibration bed, air-blowing bed or in a magnetic field^8,10–15^, and simulations about driven 2D and 3D systems^16–18^. In particular, translational motions in driven granular gases have been well studied, which usually exhibit non-Gaussian distributions of translational displacements^7,9–12^ and occasionally exhibit Gaussian distributions in 2D vibrating granular gas in microgravity^8^. However, the rotational motions are mainly studied in simulations^17–19^ and experimental measurements are limited^7,8,10,15^.

The motion of a single particle is important for understanding the collective motion of many granular particles but remains poorly understood. Single granular particle can exhibit interesting motions without external driving forces, such as the Euler disk with a faceted edge^20^ or a ring^21^ rolling on a table. A bouncing droplet on its self-activated surface wave shows nonlinear and even quantum-like behaviors^22,23^. For a single particle driven on a vertically vibrating stage, the motion in the xy plane is dominated by friction and collisions and thus difficult to model and predict in theory or simulation. Hence it is mainly studied experimentally including the motions of a dimer^24,25^, trimer^26^, uniform rod^27,28^, asymmetric rod^29–31^ and polar disk with a non-uniform mass density^32^. When their shape or mass distribution is asymmetric, the translational motion can become self-propulsive^26,30–32^. The rotational motion has been measured for non-self-propelled single rod^28^, asymmetric particles with a complex shape^33,34^, and numerically studied for chiral granular motors^35,36^.

For a single isotropic particle such as disk or sphere driven on a vibration stage, only the vertical motion of a single sphere has been studied^37^. The motion along the vertical (z) direction mainly depends on its collision with the substrate and thus is easier to predict theoretically than the motion in the horizontal (xy) plane which is dominated by friction and collisions. The collision frequency along the z direction is described by the bouncing ball model, which was proposed by Enrico Fermi for describing cosmic rays accelerating and bouncing between moving clouds of hydrogen gases in interstellar space^38^. The model also has important applications in nonlinear physics and engineering^39^. By analyzing the collision sound, the bouncing-ball experiment showed that the motion of a sphere is chaotic in the z direction along gravity, while the motion in the xy plane was suppressed by using a slightly concave substrate^40^. Although the translational motion of a single sphere has not been studied on a vibration stage, it has been measured on an air-blowing bed which exhibits a range of thermal equilibrium behaviors^2^. Here we found that a single disk driven by a vibration bed exhibits strong non-equilibrium behaviors.

A diffusion is characterized by the static probability distribution function (PDF) of spatial displacements and the dynamic mean-square displacement (MSD) as a function of time. Particles in thermal equilibrium systems usually exhibit normal diffusive motion, i.e. Gaussian PDF and Fickian diffusion whose MSD is proportional to time. In a complex environment, however, it is often non-Gaussian for short time steps^41,42^. In a non-equilibrium system, a particle can exhibit a non-Gaussian distribution of displacements even at long time scales^43^ and non-Fickian diffusion. Anomalous non-Fickian diffusion and non-Gaussian PDF exist widely in physical and biological systems^44^, such as flows through disordered media^45^, near-ballistic motion of chromosomal loci^46^, subdiffusion in membrane permeation of small molecules^47^, levy flights^48^ in chaotic flows^49^ , human transports^50^, and stretched or compressed exponential distributions in granular gas^7,16–18^.

## Experimental system

**Figure 1:**
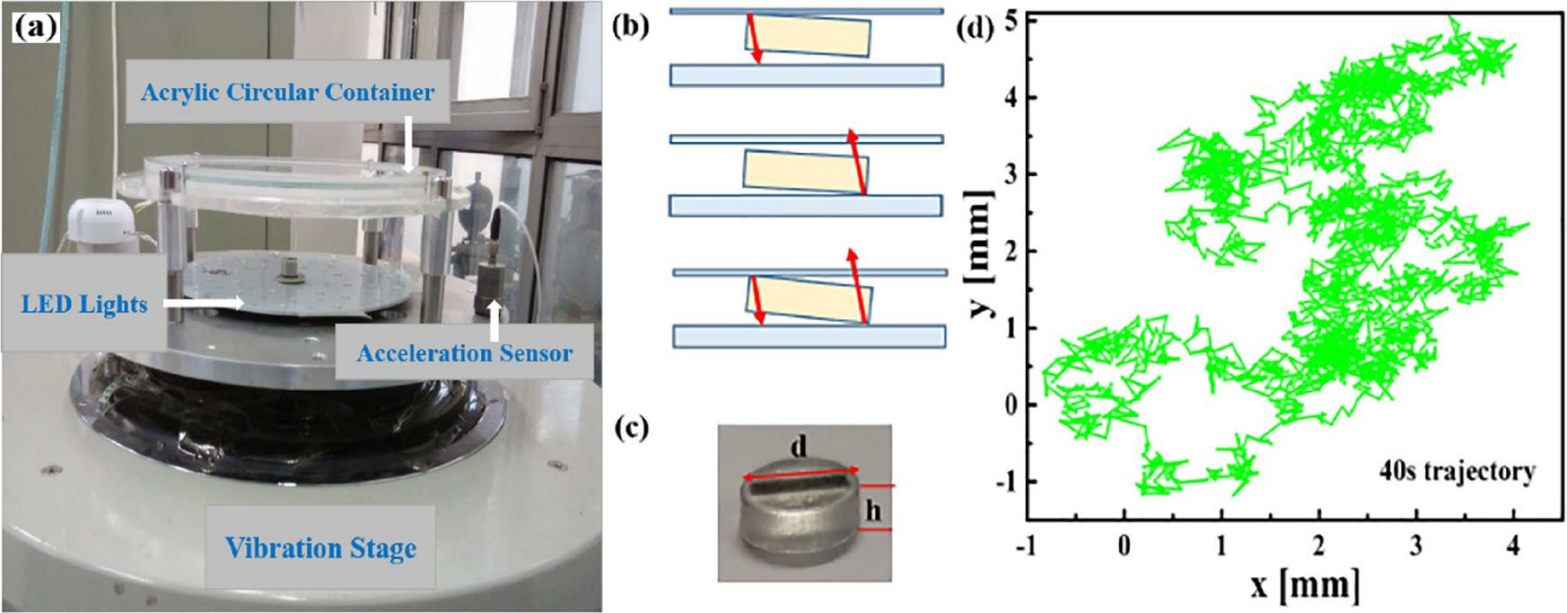
(**a**) A disk is confined in a translucent acrylic container with diameter $D=280$ mm and inner wall separation $H=4.5$ mm mounted on the vibration stage. (**b**) The disk can collide with top, bottom or both walls. (**c**) The disk has diameter $d=10$ mm, thickness $h=3.5$ mm and mass $M=2.0$ g and is made with the cartridge plastic material DurusWhite using a 3D printing system (Connex350). The black line is printed inside the disk for tracking the rotational motion in image processing and does not affect the mass distribution. (**d**) A typical 40 s trajectory of the translational motion at $\Gamma = 6.0$ ($f=50$ Hz and $A=0.6$ mm).

Here we measure the in-plane diffusion of a single disk on a vibration stage. The plastic disk is made with the cartridge plastic material DurusWhite by the 3D printing system (Connex350), and has a diameter $d=10$ mm, thickness $h=3.5$ mm, and mass $M=2.0$ g. It was placed in a circular translucent acrylic container with a diameter $D=280$ mm and inner height $H=4.5$ mm, see Fig. 1a–c. The disk collides with the two horizontal inner walls and the friction causes random in-plane translational motion and rotational motion. The quasi-2D confinement prevents the disk from flipping. The kinetic energy is much greater than the gravitational potential energy change of the disk, therefore the lid and substrate have similar effects on the disk. The electromagnetic vibration table (Zhengyi VS-1000VH-51) vibrates the object attached to it via a linear bearing to ensure the vibration is strictly vertical, and the error of control parameters $< \pm 5\%$ according to the manufacture’s calibration report. The container was firmly fixed to the vibration table via an aluminum alloy head expander by four screws, and the illumination LEDs are firmly fixed to the expander below the container (Fig. 1a). The container was leveled horizontally to an accuracy of $0.1^{\circ }$. 0.125 mm thick thin films of polyethylene terephthalate coated with conductive indium tin oxide cover both inner surfaces of the container to prevent the buildup of static electric charges from the friction between the disk and the container. The container lid is made of a fluorine-doped tin oxide conductive glass for the same reason. The stage vibrates vertically as $A\sin {(2\pi f t)}$, where *A* is the amplitude, *f* is the vibration frequency of the stage and *t* is the time. The vibration strength is described by the dimensionless acceleration

1 $$\begin{aligned} \Gamma \equiv a/g= A(2\pi f)^2/g, \end{aligned}$$

where *a* is the maximum acceleration of the stage, *g* is the free fall acceleration. Two out of the three parameters *A*, *f* and *a* are independent. In contrast to a single vibrating rod^27^ whose diffusion is dictated by $\Gamma$, we find the diffusion of the disk exhibits different behaviors under a fixed $\Gamma$. In our experiments, $f=50$ Hz, 60 Hz, 80 Hz, 100 Hz at a fixed $A=0.6$ mm, which correspond to $a= 6.0~g$, 8.7 *g*, 15.5 *g*, and 24.0 *g* respectively (see Supplementary Movies 1-4). Thus, a higher frequency represents a stronger driving force, in accordance with our observed stronger motions at higher *f*. We observe similar motions of the disk at different positions on the stage, indicating a uniform vibration. The LED lights between the container and vibration stage illuminate the container uniformly. When the acceleration exceeds the critical value of 4 g, the disk starts to move. The motion of the disk is recorded by a CMOS (complementary metal oxide semiconductor) camera (Lunemera lt225) placed above the container, at 150 frames/s for about 5-10 min before the disk hits the boundary of the container. The center of mass of the disk in each frame is measured by the standard particle-tracking algorithm for tracking spheres^51^. The black line on the disk (Fig. 1b) is tracked by a rectangular mask, which gives the orientation of the disk.

The disk mainly rolls on the top and bottom plates without slipping and the restitution coefficient $e_r$ associated with the occasional collisions are relatively less important than friction. Both the friction coefficient and the restitution coefficient between the disk and the conductive film covered on the acrylic lid and substrate have been measured (see Supplementary Information). The measured static friction coefficient $\mu = 0.157 \pm 0.001$. As the restitution coefficient $e_r$ depends on particle shape, collision velocity, collision direction, etc^26^, we mimicked the motion of disk on the vibration stage and measured $e_r$ using an acoustic stopwatch provided by App Phyphox^52^. The measured $e_r$ can be well fitted by $e_r \propto v^{-\frac{4}{5}}$, in contrast to $e_r \propto v^{-\frac{1}{5}}$ for stainless steel spheres^53^ in the similar speed regime. At the low speed $v = 0.59$ m/s, a velocity closer to our experimental condition, $e_r = 0.93$, which is close to the restitution coefficient 0.934 between two acrylic spheres reported in ref. ^54^.

## Results

### Probability distribution function of displacements

**Figure 2:**
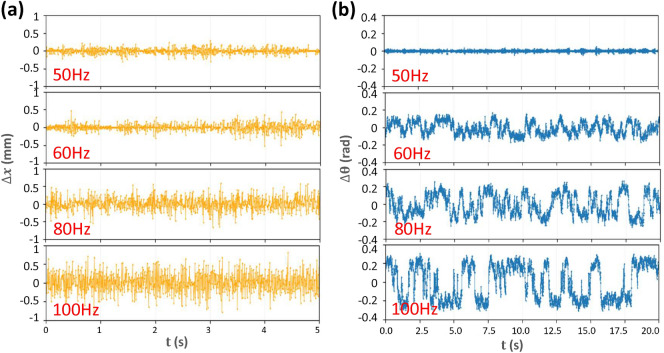
(**a**) Translational and (**b**) angular displacements per 1/150 s at the driving frequencies $f=50$ Hz, 60 Hz, 80 Hz, and 100 Hz, corresponding to $\Gamma = 6.0, 8.7, 15.5$, and 24.0 respectively. $A=0.6$ mm.

Figure 2 shows the typical translational and angular displacements. The translational motion is isotropic along the x and y directions, thus Fig. 2a shows the distributions of displacements along both x and y directions. Rotations often maintain in the same direction for a while and such persistent time is substantially increased at higher *f* (Fig. 2b). The angular displacements for the clockwise and counterclockwise rotations have similar magnitude (Fig. 2b).

**Figure 3:**
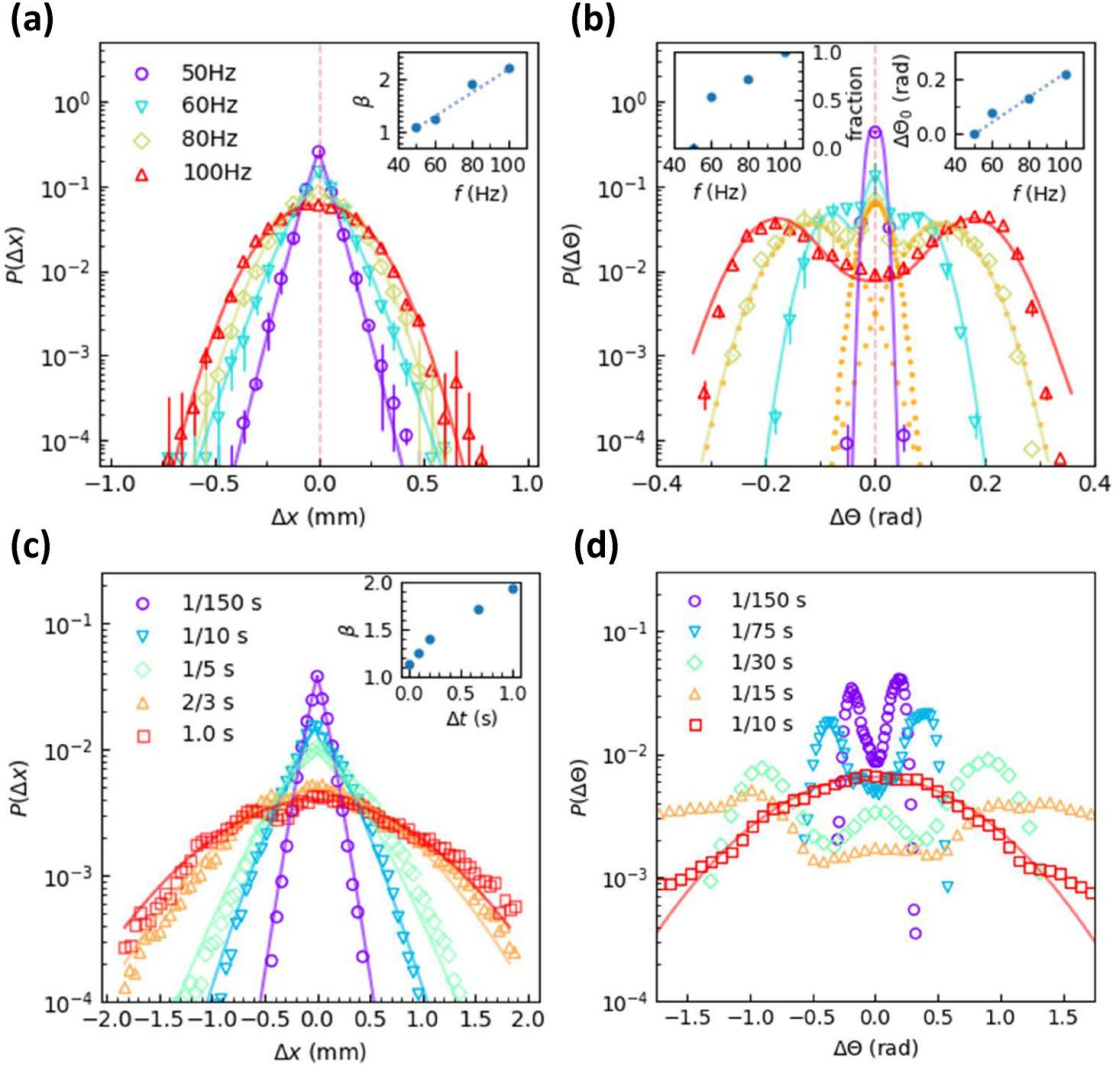
PDFs of (**a**) translational and (**b**) rotational displacements per $\Delta t =1/150$ s at $A=0.6$ mm and $f=50$ Hz, 60 Hz, 80 Hz, and 100 Hz, corresponding to $\Gamma = 6.0, 8.7, 15.5$, and 24.0 respectively. The angular displacement $\Delta \theta$ describes the disk’s rotation in the xy plane, not the tilting angle limited by the lid as shown in Fig. 1b. (**a**) PDFs fitted by the compressed exponential functions $f(x)=Ae^{-Bx^\beta }$ (curves) with the $\beta$ shown in the inset. (**b**) Each PDF is fitted by a Gaussian function peaking at $\theta =0$ and two Gaussian functions peaking at $\pm \Delta \theta _0$ (curves). As an example, the dashed curves represent the three Gaussian distributions peaking at $\Delta \theta =0, \pm 0.12$ for $f=80$ Hz, corresponding to the inactive rotation, clockwise and counterclockwise active rotations, respectively. $\Delta \theta _0$ is shown in the right inset. The fraction of active mode measured from the three Gaussian fits is shown in the left inset. (**c**) PDFs of translational displacements under 60 Hz with $\Delta t = 1/150$ s, 1/10 s, 1/5 s, 2/3 s, and 1.0 s fitted by the compressed exponential functions (curves) with the $\beta$ shown in the inset. (**d**) PDFs of rotational displacements under 100 Hz with $\Delta t = 1/150$ s, 1/75 s, 1/30 s, 1/15 s, and 1/10 s with $\Delta t = 1/10$ s fitted by a Gaussian function (curves). The inverse uncertainty is used as the weight of each data point in all the fittings.

The PDFs of translational displacements are symmetric around $x=0$ and each branch follows the compressed exponential function $f(x)=Ae^{-Bx^\beta }$ as shown in Fig. 3. The fitted $\beta$ linearly increases with *f* (Fig. 3a inset). The distribution is close to exponential (i.e. $\beta =1$) at 50 Hz and Gaussian (i.e. $\beta =2$) at 80 Hz. $\beta =1, 2$ are commonly observed in various diffusions^41–43^, while $\beta >2$ is uncommon. The compressed exponential distribution (i.e. $\beta >1$) is common in granular gas, but has not been observed in the motion of single particle. Interestingly, the PDFs of rotational displacements exhibit a sharp peak at $x=0$ at low frequencies, three peaks at $0,\pm \Delta \theta _0(f)$ at intermediate frequencies, and two peaks at $\pm \Delta \theta _0(f)$ at high frequencies (Fig. 3b). The symmetric peak position $\theta _0$ linearly increases with *f* (Fig. 3b inset).

The sharp peak of the PDF at $x=0$ in Fig. 3b reflects an inactive state that the disk does not rotate much. At low frequencies, i.e. low driving forces, the disk lies on the substrate with a 0$^\circ$ tilting angle so that the friction suppresses the rotation. As *f* increases, i.e. $\Gamma$ increases, more rotations are activated (left inset of Fig. 3b). In the active mode, the disk persistently rotates either clockwisely or counter-clockwisely, resulting two peaks symmetrical around $\Delta \theta =0$ (Fig. 3b). These persistent rotations last about one second which can be directly observed by eyes (see Supplementary Movie 3). Such a persistent rotation is much longer than the vibration period, indicating that the disk-wall interaction during each vibration period usually does not strongly affect the rotation. The tilted disk may wobble as a disk^55^, a Euler disk^20^ or a ring^21^ rolling on a table with a certain procession frequency $\Omega \propto \sqrt{g/d}$ where *d* is the particle diameter. In fact, $\Omega \propto \sqrt{g/d}$ can be derived from the dimensional analysis, and the complicate prefactor depends on the titling angle, particle shape, air friction, and slipping condition^55^. The disk diameter $d=1$ cm is much larger than the gap between the disk and walls ($4.5~\text {mm}-3.5~\text {mm}=1.0$ mm), thus the opposite rims of a slightly tilted disk can be in contact with both the top and bottom walls. The disk is likely to roll on the two walls without collisions to maintain the persistent rotation, thus $\Omega \propto \sqrt{a/d}$ should similarly hold for our case. Indeed, we confirm $\Omega \propto \sqrt{a}$ in Fig. 3 right inset because the acceleration $a\propto f^2$ as shown by Eq. (1). When the disk loses contact with walls, the subsequent collision tends to change the rotational direction. This explains the bimodal peaks in Fig. 3. To our knowledge, the bimodal distribution of rotational displacements has not been observed in other diffusion systems or granular systems. When the chosen time step increases, the PDFs become closer to Gaussian as shown in Fig. 3c, d, in accordance with the central limit theorem.

### Energy distribution

**Figure 4:**
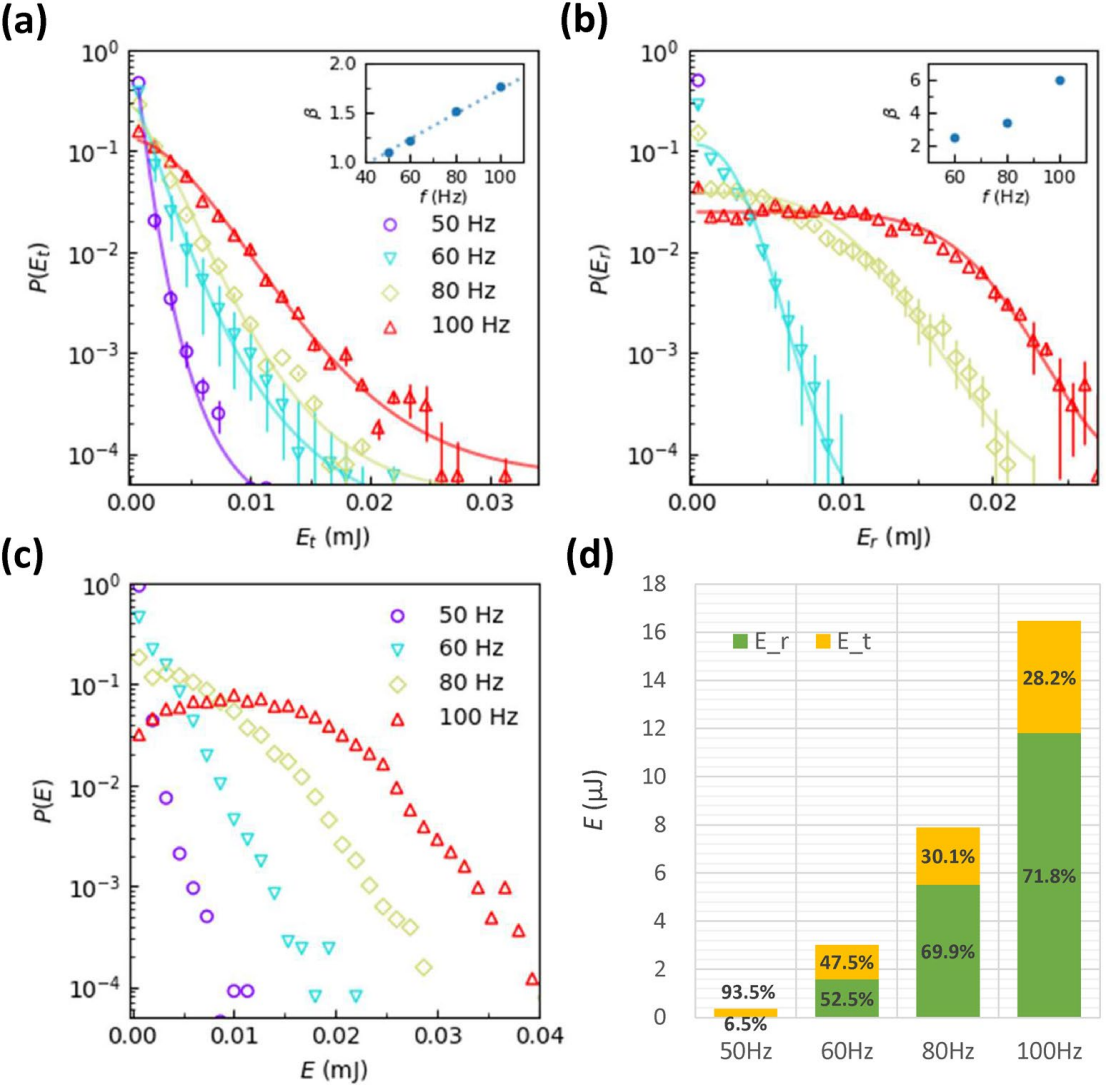
The estimated (**a**) translational and (**b**) rotational kinetic energy distributions fitted by $f(x)=Ae^{-Bx^\beta }$ (curves) with $\beta$ shown in the insets. (**c**) The total kinetic energy distributions. (**d**) The total kinetic energies labeled with the fraction of translational and rotational energies at different *f*. $A=0.6$ mm. $f=50$ Hz, 60 Hz, 80 Hz, and 100 Hz, corresponding to $\Gamma = 6.0, 8.7, 15.5$, and 24.0.

An air-blowing bed can ‘thermalize’ granular particles^2^. For example, the effective temperature^56^ of granular particles on the air-blowing bed satisfies many relations in equilibrium statistical mechanics^2^. Here we find that the translational kinetic energy $E_\text {t}=\frac{1}{2} m v^2$ and rotational kinetic energy $E_\text {r}=\frac{1}{2} I \omega ^2$ in 5 experimental trials do not exhibit exponential distributions (Fig. 4a, b), i.e. disobeying the Boltzmann distribution. The moment of inertia $I=\frac{1}{2} m(\frac{d}{2})^2$. The speed $v=\Delta r/\Delta t$, and the angular speed $\omega =\Delta \theta /\Delta t$. The time step $\Delta t = 1/150$ s which is shorter than the vibration periods, hence the translational speed can be roughly estimated. The angular speed can be more accurately measured because the disk persistently rotates along one direction for a much longer period than $\Delta t$. Both the translational and rotational energy distributions can be well fitted by compressed exponential distributions (Fig. 4a, b). The energy distribution is close to exponential, i.e. Boltzmann, distribution only for translational motion at low *f*. The distribution of the total energy in Fig. 4c is exponential at low *f* and exhibits a dip at high *f*. By contrast, the distribution of total kinetic energy of a vibrating rod always has a dip at zero energy and an exponential tail at high energy under different accelerations^27^.

The translational energy dominates at low *f* and the rotational energy dominates at high *f* (Fig. 4d), which deviates from the thermal equilibrium behavior of $E_\text {t}: E_\text {r}=2:1$ according to the equipartition theorem. It is however consistent with the observation that disk-wall collisions can easily change the direction of the translational motion (Fig. 2a) but not the direction of rotational motion, especially at high *f* (Fig. 2b). Since the collisions interrupt rotations less, the rotational energy occupies a higher fraction in the total energy at high *f*.

### Mean squared displacement

**Figure 5:**
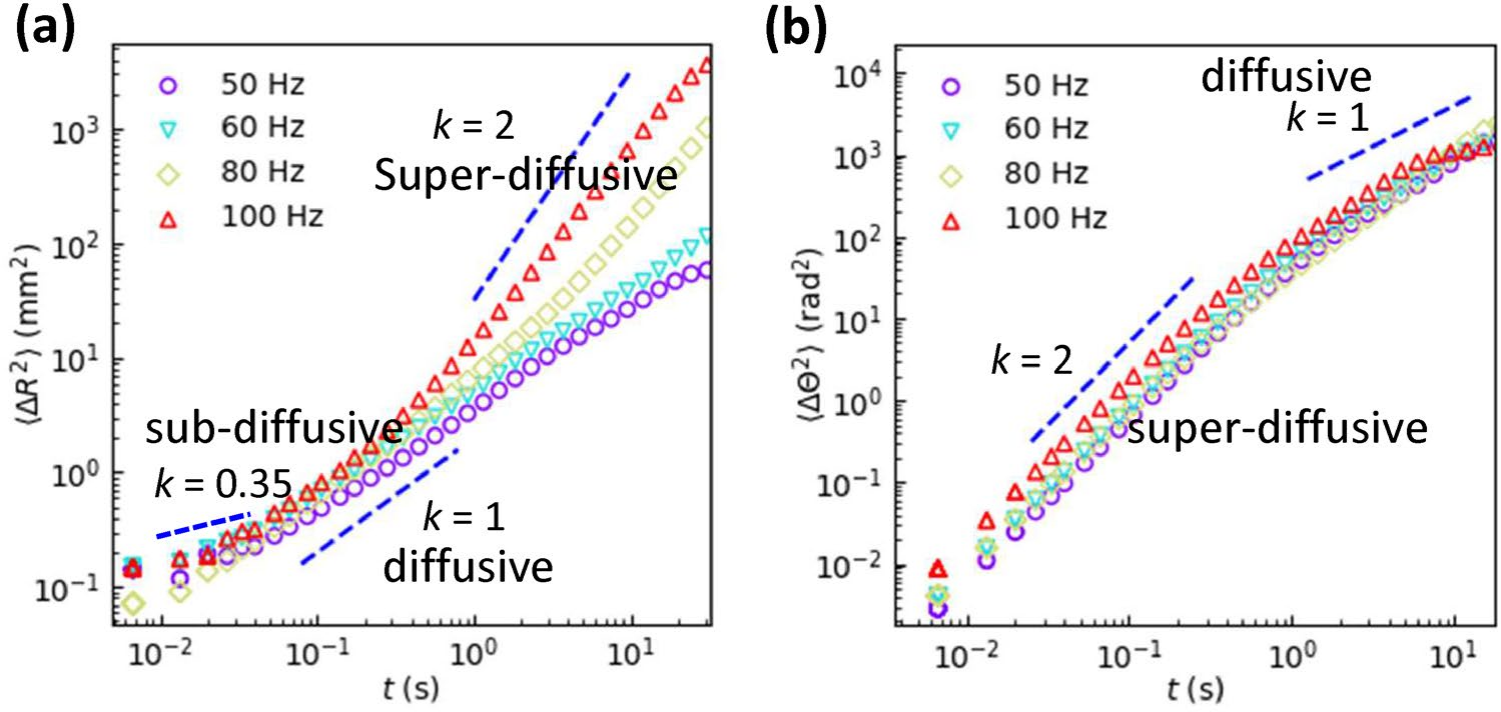
(**a**) Translational MSDs and (**b**) Rotational MSDs $\propto t^k$ under $A=0.6$ mm and $f=50$ Hz, 60 Hz, 80 Hz, and 100 Hz, corresponding to $\Gamma = 6.0, 8.7, 15.5$, and 24.0.

**Figure 6:**
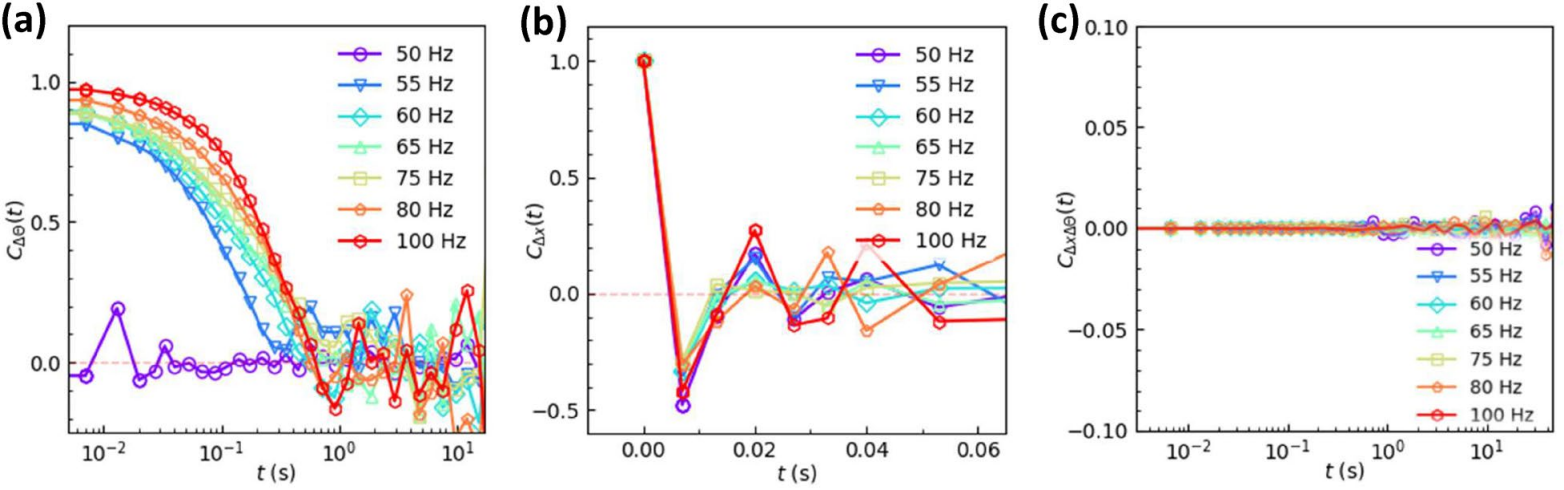
Time autocorrelation functions *C*(*t*) of the (**a**) translational displacements $\Delta x$ and (**b**) angular displacements $\Delta \theta$ under various *f*. (**c**) The cross-correlations of the translational motion and rotational motion. $A=0.6$ mm. $f=50$ Hz, 60 Hz, 80 Hz, and 100 Hz, corresponding to $\Gamma = 6.0, 8.7, 15.5$, and 24.0. $\Delta t =1/150$ s.

The diffusion is characterized by the mean squared displacement $\mathrm {MSD}\equiv \langle \Delta r^2(t)\rangle =\langle (r(t+t_0)-r (t_0))^2\rangle$, where *r*(*t*) is the center position of the disk at time *t*. $\langle \rangle$ averages over all initial times $t_0$ in each trajectory and all 5 experimental trials. We found that $\mathrm {MSD} \sim t^k$ with different *k* in different time regimes, i.e. non-Fickian diffusions. In Fig. 5a, the single disk in our system appears to roll around a center at $t<0.05$ s which leads to a caging-like effect, and this center randomly diffuses in a longer time regime. The single disk in our system has no caging environment, indicating that the interactions can provide a similar caging effect. Indeed, interactions tend to change the direction of the translational velocity as shown by the negative speed correlations at short *t* in Fig. 6b. For the angular $\text {MSD}_\theta \equiv \langle \Delta \theta ^2(t)\rangle =\langle (\theta (t+t_0)-\theta (t_0))^2\rangle$ shown in Fig. 5b, their slopes $>1$ at short times ($t<1$ s) and becomes 1 at long times, suggesting a crossover from short-time ballistic rotations ($k=2$) to long-time diffusive ($k=1$) rotations. The ballistic rotation ($k=2$) is a special case of superdiffusion ($k>1$) and is in accordance with the persistent rotation along one direction for about 1 sec before the direction of rotation flips and the positive correlations of the angular speed in Fig. 6a. A higher frequency corresponds to a stronger driving force and hence faster diffusion as shown in Fig. 5.

### Displacement correlations

For Brownian motion, displacements are white noise without memories, i.e. the time auto-correlations are zero. Here we find interesting displacement correlations for the driven disk that are distinct from those of Brownian motion. The time autocorrelation functions $C_{\Delta x} =\langle \Delta x(t_0) \cdot \Delta x(t_0+t)\rangle$ for translational displacements oscillate between positive and negative values at short time and rapidly decay to zero in less than 0.1 s (Fig. 6b), indicating that the displacement tends to change direction by collisions and lost memory at 0.1 s, in accordance with the crossover from subdiffusive motion to diffusive motion at about 0.1 s in the MSDs of Fig. 5a. The interaction between the edge of the disk and the substrate tends to reverse the center-of-mass speed, which effectively randomizes the translational motion. The interaction points towards the center of the disk which should not strongly affect the rotation. The time autocorrelation functions $C_{\Delta \theta } =\langle \Delta \theta (t_0) \cdot \Delta \theta (t_0+t)\rangle$ for rotational displacements are positive and decay to zero over 0.1 s to 10 s (Fig. 6a). Such memory time of the rotation is in accordance with the crossover from superdiffusive motion to diffusive motion at about 0.1 to 10 s shown in the MSDs of Fig. 5b. The memory time of rotation increases with *f*, which is consistent with the longer persistent time at high *f* in Fig. 2b. The correlation between translational and rotational motions of a single driven particle has been studied for a rod on a vibrating stage; its translational kinetic energy in the z direction and kinetic energy of rotation are weakly correlated at short time^28^; its translational and rotational energies in the vertical plane are strongly correlated^27^.

The coupling between the translational motion and the rotational motion can be quantified by a dimensionless cross-correlation function $C_{\Delta x\Delta \theta }=(\langle \Delta x\Delta y \sin {\theta }\rangle ) / (\Delta x^2+\Delta y^2)$, where $\Delta x$ is the displacement during $[t_0, t_0+t]$ and $\theta$ is the orientation at $t_0+t/2$^57^. The measured $C_{\Delta x\Delta \theta }$ in Fig. 6c are close to zero, indicating no coupling between the translational and rotational motions. By contrast, $C_ {\Delta x\Delta \theta }$ is non-zero for the diffusion of an ellipsoid in both passive liquid^57^ and active liquid^58^, indicating that the translational-rotational coupling is sensitive to the particle shape, but not to driving forces. Besides, the interparticle collisions will produce non-zero translation-rotation correlations in granular gases^59^, in contrast to the zero correlations for a single particle in this study.

### Power spectrum

**Figure 7:**
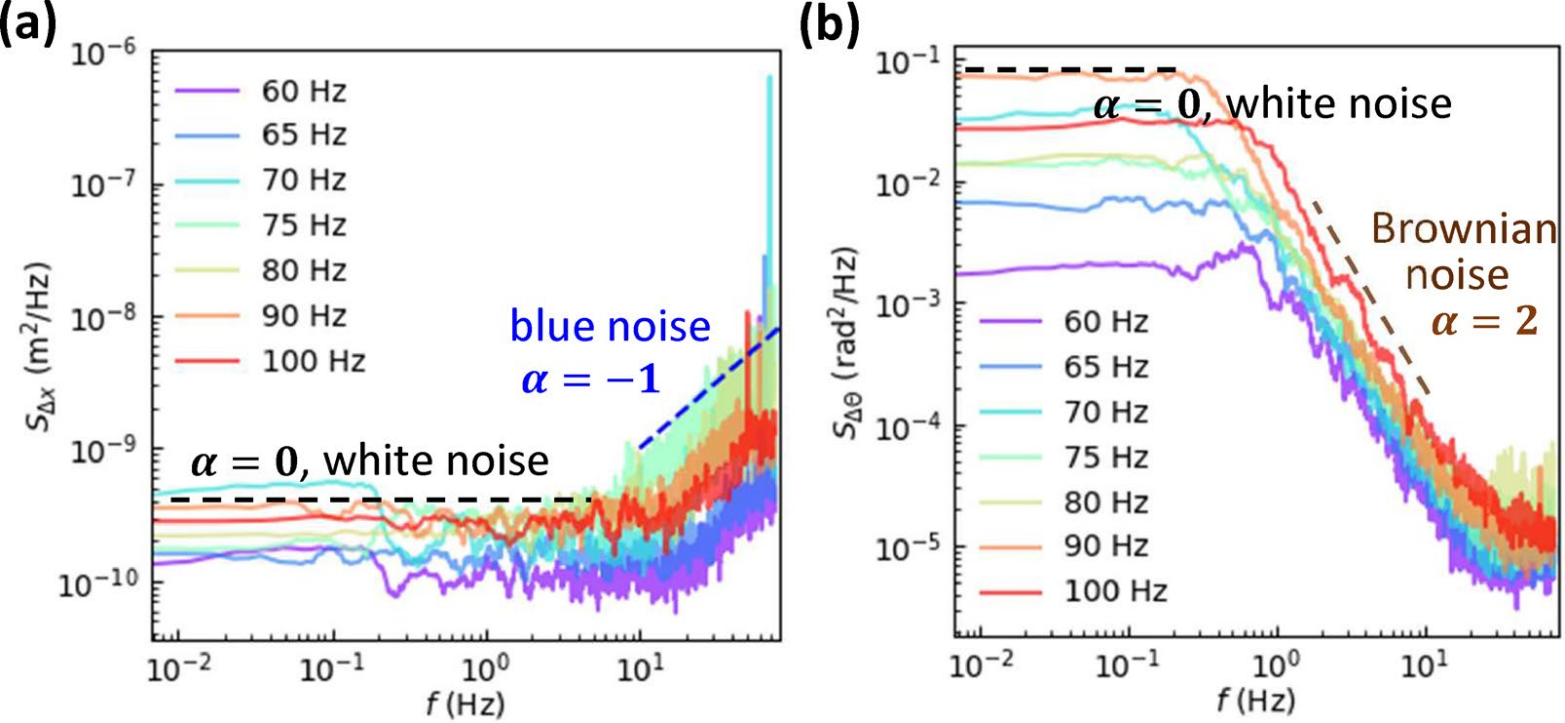
Power spectral densities of (**a**) $\Delta x$ and (**b**) $\Delta \theta$ at various *f* from 60 Hz to 100 Hz at $A=0.6$ mm, corresponding to $\Gamma$ varying from 8.7 to 24.0. $\Delta t =1/150$ s.

The power spectrum, or the power spectral density, $S_{x}(f)=\left| \int _0^{t_\text {tot}}e^{-2\pi ift}x(t)\,dt\right| ^2/(2\pi t_\text {tot})$ provides another angle from which to characterize a time series *x*(*t*) in the frequency domain. $v_x^2\propto E_\text {t}$ and $\omega ^2\propto E_\text {r}$, hence $S_{\Delta x}$ and $S_{\Delta \theta }$ in Fig. 7 reflect the kinetic energy distributions in the frequency domain. In $S(f) \sim f^{-\alpha }$, the exponent $\alpha$ characterizes the color of the noise. $\Delta x$ exhibits white noise ($\alpha =0$) at low frequencies and blue noise ($\alpha =-1$) at high frequencies (Fig. 7a), while $\Delta \theta$ are white noise at low frequencies and Brownian noise ($\alpha =2$) at high frequencies (Fig. 7b). Although the $\alpha =-1$ regime only spans 1 order of magnitude, it is robust for different trials of experiments which lends confidence to the blue noise. Interestingly, $S_{\Delta \theta }$ is similar to the power spectrum of the angular velocity of a probing blade embedded in a shaking granular medium^60^, suggesting that the rotation power spectra are insensitive to granular density.

## Discussion and conclusion

We measure the motion of a single uniform-density disk driven on a vibration bed for the first time. The translational displacements are isotropic in the xy plane and exhibit compressed exponential distributions whose $\beta$ increases with frequency. The compressed exponential distribution is uncommon in diffusion problems, and has been reported in granular gases^7,16–18^. The rotational displacements exhibit unimodal, trimodal and bimodal distributions and each peak can be fitted by a Gaussian function. The sharp peak at $\Delta \theta =0$ and two broad peaks at $\pm \Delta \theta _0$ correspond to inactive mode, clockwise and counterclockwise active modes respectively. The persistent rotation along one direction in the active mode reflects disk rolling on both walls without collisions. Such processional angular speed $\Omega \propto \sqrt{a/d}$ which is partly confirmed by our measured $\Omega \propto \sqrt{a}\propto f$. The MSDs show that the translational motion is subdiffusive at short times and superdiffusive at long times, while the rotational motion is superdiffusive at short times and diffusive at long times. Their crossover time scales correspond to the memory times shown in the auto-correlations of displacements. The translational and rotational motions have no correlation, indicating that the previous observed translation-rotation correlations in granular gases^59^ arise from inter-particle collisions. The rotational displacements are positively correlated over a long period time (about 1 s) especially at high frequencies, while the translational displacements are negatively correlated at short times. These indicate that the disk slightly tilt and roll on the two walls most of the time. Such rolling does not change the rotational direction, but tends to change the direction of translational motion every half period and produce negative correlations for translational displacements at short times.

We find that most of the input energy is in the translational motion at low frequencies and in the rotational motion at high frequencies. This violation of the equipartition theorem is a critical feature of non-equilibrated systems. By contrast, particles driven by an air-blowing bed follow thermal equilibrium behaviors including those described by the equipartition theorem^2^. Therefore, the vibration bed is quite different from the air bed. Our observation that the stronger rotational motion than the translational motion for a single particle at high *f* could deepen the understanding of multiple-particle systems. For example, the stronger translational motion in denser granular systems on a vibration stage^11^ could be explained as more inter-particle collisions at a higher density transferring more energy from rotation motion to translational motion^11^. If we only consider the translational motion, as we may in the absence of rotational information, the motion of particles in ref. ^11^ could appear abnormal.

## Supplementary information

Supplementary material 1(avi 772 KB)

Supplementary material 2 (mp4 1040 KB)

Supplementary material 3 (mp4 1556 KB)

Supplementary material 4 (mp4 1275 KB)

Supplementary material 5 (pdf 254 KB)
