# Supplementary material for: Dynamics of a vibration-driven single disk
Source: Sci Rep. 2021 Aug 16;11:16561. doi: 10.1038/s41598-021-95672-6 (PMC8367964; doi:10.1038/s41598-021-95672-6)
Supplement: Supplementary file 5 — Supplementary material 5 (pdf 254 KB) [file 41598_2021_95672_MOESM5_ESM.pdf]

Dynamics of a vibration-driven single disk

Supplementary Information

Liyang Guan, Li Tian, Meiying Hou, Yilong Han

Parameter measurement

Friction coefficient

The friction coefficient is measured from the setup shown in Fig. S1. A mass is stuck on the top of the center of four disks (Fig. S1 inset), with a total mass M_1 = 158.2 g. The mass and four disks are placed on a horizontal acrylic plate coated with the conductive film. A string attaches M_1 at one end and a small tray at the other end. The string is placed over a pulley so that adding enough weights into the tray can vertically pull the string in gravity and drive M_1 sliding horizontally (Fig. S1). The position of the pulley is well adjusted so that the string between the mass and pulley is horizontal. When the mass of the tray and the weights in it reaches the critical value M_2, M_1 can maintain stationary for a while and then starts to slide. We find the averaged M_2 = (24.8 ± 0.2) g from 10 trials of measurements when it can maintain the system stationary for > 10 min before it starts to move. Thus the static friction coefficient $\mu = M_2/M_1 = 0.157 \pm 0.001$.

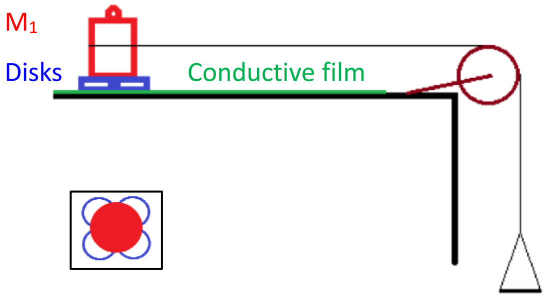

Fig. S1 The setup for measuring the friction coefficient. Inset: Top view of the mass and four disks.

Restitution coefficients

Restitution coefficient e_r is the ratio of the speeds before and after collision. It's well-known that e_r depends on various factors such as particle shape, collision velocity and collision direction (Ref. 26). For example, different collision points of an anisotropic object produce different deformations, that is, different energy dissipations and e_r. Nevertheless, our disk mainly rolls on the top and bottom plates without slipping and e_r associated with the occasional collisions are relatively less important than friction. We measure e_r between the disk and the acrylic substrate coated with a transparent conductive film (Simihui ITO Conductive Film PET Film; product description: 150ohm, 125um*406mm*3.3m). Such coated substrate is the same as the coated inner surface of the container on the vibration stage. To mimic the disk on the vibration stage, the horizontally orientated disk is released from a small height and then bounces several times. The coefficient is calculated by $e_r = \frac{v_n}{v_{n-1}} = \frac{g \cdot T_n}{g \cdot T_{n-1}} = \frac{T_n}{T_{n-1}}$. T_n is the interval between the n'th and the (n - 1)'th collisions, which is automatic measured by an automatic acoustic stopwatch in the App Phyphox on a smartphone (Ref. 52) with a resolution of 0.01 ms. v_n is the speed. Since e_r depends on the collision speed, we release the disk at different heights (i.e. different collision speed *v*). The disk keeps parallel with the substrate only in the first few collisions and becomes rolling on the substrate like a Euler disk soon after, thus only the first few collisions are used for the measurement of e_r. The measured e_r in

20 trials of experiment can be well fitted by $e_r \propto v^{-\frac{4}{5}}$ (Fig. S2).

Fig. S2 The restitution coefficient e_r at different collision speeds $v = \sqrt{2gh}$ measured from different height of release h.

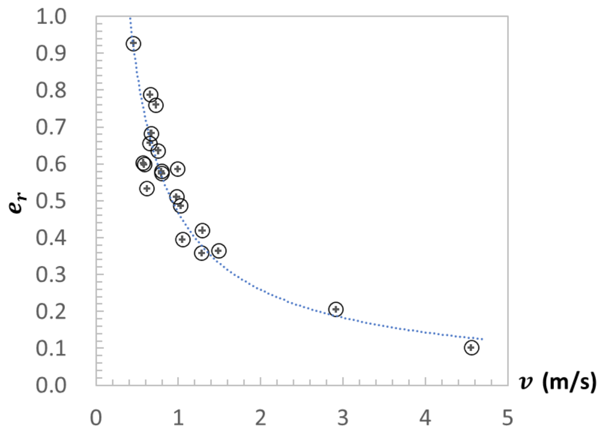

Movies

- Movie 1: The disk moving on the vibration stage at 50 Hz with amplitude 0.6 mm. Only the black line inside the disk can be seen under the intense illumination light. The disk can be seen in movie 2.
- Movie 2: The disk moving on the vibration stage at 60 Hz with amplitude 0.6 mm. The black line is printed inside the disk for tracking the position and orientation.
- Movie 3: The disk moving on the vibration stage at 80 Hz with amplitude 0.6 mm. Only the black line inside the disk can be seen under the intense illumination light. The disk can be seen in movie 2.
- Movie 4: The disk moving on the vibration stage at 100 Hz with amplitude 0.6 mm. Only the black line inside the disk can be seen under the intense illumination light. The disk can be seen in movie 2.
